# Anti-*Candida albicans* Activity of Thiazolylhydrazone Derivatives in Invertebrate and Murine Models

**DOI:** 10.3390/jof4040134

**Published:** 2018-12-12

**Authors:** Lana Ivone Barreto Cruz, Larissa Ferreira Finamore Lopes, Felipe de Camargo Ribeiro, Nívea Pereira de Sá, Cleudiomar Inácio Lino, Nagendran Tharmalingam, Renata Barbosa de Oliveira, Carlos Augusto Rosa, Eleftherios Mylonakis, Beth Burgwyn Fuchs, Susana Johann

**Affiliations:** 1 Departamento de Microbiologia, Instituto de Ciências Biológicas, Universidade Federal de Minas Gerais, Avenida Presidente Antônio Carlos, 6627, Pampulha, Belo Horizonte—Minas Gerais 31270-901, Brasil; lanabarretocruz@gmail.com (L.I.B.C.); lafinamore@gmail.com (L.F.F.L.); niveasap@gmail.com (N.P.d.S.); carlrosa@icb.ufmg.br (C.A.R.); 2 Departamento de Biociências e Diagnóstico Bucal, Instituto de Ciência e Tecnologia de São José dos Campos—UNESP, Av. Francisco José Longe, 777, Jardim São Dimas, São José dos Campos—São Paulo 12245-000, Brasil; felipe_c_ribeiro@hotmail.com; 3 Department of Molecular Genetics and Microbiology, Division of Infectious Diseases, Stony Brook University, 150 Life Science Building, Stony Brook, NY 11794, USA; 4 Departamento de Produtos Farmacêuticos, Faculdade de Farmácia, Universidade Federal de Minas Gerais, Belo Horizonte—Minas Gerais 31270-901, Brasil; inaciol@hotmail.com (C.I.L.); renatabo.ufmg@gmail.com (R.B.d.O.); 5 Division of Infectious Diseases, Rhode Island Hospital, Alpert Medical School, and Brown University, Providence, RI 02903, USA; nagendran_tharmaligam@brown.edu (N.T.); emylonakis@lifespan.org (E.M.); helen_fuchs@brown.edu (B.B.F.)

**Keywords:** *Candida albicans*, thiazolylhydrazone derivatives, antifungal

## Abstract

Candidiasis is an opportunistic fungal infection with *Candida albicans* being the most frequently isolated species. Treatment of these infections is challenging due to resistance that can develop during therapy, and the limited number of available antifungal compounds. Given this situation, the aim of this study was to evaluate the antifungal activity of four thiazolylhydrazone compounds against *C. albicans*. Thiazolylhydrazone compounds **1**, **2**, **3**, and **4** were found to exert antifungal activity, with MICs of 0.125–16.0 μg/mL against *C*. *albicans*. The toxicity of the compounds was evaluated using human erythrocytes and yielded LC_50_ > 64 μg/mL. The compounds were further evaluated using the greater wax moth *Galleria mellonella* as an in vivo model. The compounds prolonged larval survival when tested between 5 and 15 mg/kg, performing as well as fluconazole. Compound **2** was evaluated in murine models of oral and systemic candidiasis. In the oral model, compound **2** reduced the fungal load on the mouse tongue; and in the systemic model it reduced the fungal burden found in the kidney when tested at 10 mg/kg. These results show that thiazolylhydrazones are an antifungal towards *C. albicans* with in vivo efficacy.

## 1. Introduction

Systemic fungal infections are responsible for high mortality and morbidity, constituting a serious public health problem [1]. Among the opportunistic fungi, *Candida albicans* is responsible for most fungal infections [2,3] and has emerged as a major public health problem during the past two decades [4].

*C. albicans* is a commensal microorganism to the human body, present in the gastrointestinal tract, oral, and vaginal microbiota. However, factors can arise that alter host defense mechanisms and contribute to *C. albicans* transition from commensal to pathogenic [5]. As pathogenic fungi, *C. albicans* can cause superficial infections, such as oral or vaginal candidiasis, or in more serious cases, systemic candidiasis with risk of death [6]. Systemic antifungal agents include amphotericin B, the azoles (fluconazole, itraconazole, voriconazole), and echinocandins. Reports of resistance to these therapeutic agents have been on the rise, which highlights the need for new antifungals [7].

Thiazolylhydrazone derivatives represent a promising class of bioactive compounds, which have shown antifungal activity against important clinical fungal species. The antifungal activity of these molecules against *C. albicans*, *Candida krusei*, *Candida parapsilosis*, *Candida glabrata*, *Cryptococcus neoformans*, and *Cryptococcus gattii* has been reported [8,9,10]. In addition to the attractive antifungal activity, these derivatives have exhibited low toxicity towards vero, human embryonic, and liver cells [8,9,10].

The objective of the present study was to evaluate the activity of four heterocyclic thiazolic compounds. The study examined antimicrobial inhibition, compound toxicity, and efficacy within a model host.

## 2. Materials and Methods

### 2.1. Compounds

**Figure 1:**
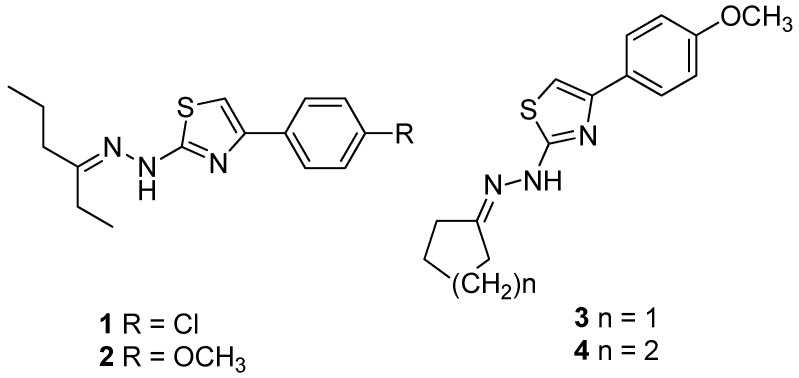
Chemical structure of thiazolylhydrazones **1**–**4**.

Thiazolylhydrazone derivatives 2-((2-(hexan-3-ylidene)hydrazino)-4-(4-chlorophenyl)-thiazole (1), 2-((2-(hexan-3-ylidene) hydrazino)-4-(4-methoxyphenyl)-thiazole (2), 2-((2-cyclohexylidene)hydrazino)-4-(4-methoxyphenyl)-thiazole (3), and 2-((2-cyclopentylidene)hydrazino)-4-(4-methoxyphenyl)-thiazole (4) (Figure 1) were synthesized according to methodology previously described by [10]. Fluconazole and amphotericin B were purchased from Sigma Aldrich, St. Louis, MO, USA.

### 2.2. Microbial Strains and Inoculum Preparation

In this study we used the references strains *C. albicans* (CAN14), *C. albicans* (DAY185), *C. dubliniensis* (ATCC MYA-646), *C. krusei* (*Pichia kudriavzevii*) (ATCC 6258), *C. glabrata* (ATCC 90030), *C. parapsilosis* (ATCC 22019), *C. tropicalis* (ATCC 13803), *Cryptococcus neoformans* (H99) and clinical isolates of *C. albicans*, *C. glabrata* and *C. neoformans* derived from Massachusetts General Hospital, MA, USA [11] and *C. parapsilosis* clinical isolates derived from Brazil [12]. All strains were stored as frozen stocks at −80 °C and sub-cultured on Sabouraud Dextrose Agar (SDA) plates at 30 °C as needed.

### 2.3. Determination of Minimum Inhibitory Concentration (MIC)

Minimum inhibitory concentration (MIC) was determined by the broth microdilution method according to the standards proposed in the CLSI M27-A3 [13]. Synthetic compounds and amphotericin B were diluted in dimethylsulfoxide (DMSO) while fluconazole was dissolved in sterile distilled water. Serial dilutions were prepared in RPMI 1640 medium supplemented with l-glutamine and buffered to pH 7.0 with 0.165 M morpholine propanesulfonic acid (MOPS). Then 50 µL of each dilution were distributed into 96 well microplates (Difco Laboratories, Detroit, MI, USA). The concentration range tested was from 0.125 to 64 µg/mL for synthetic compounds and fluconazole (FLC), and 0.06 to 16 µg/mL for amphotericin B (AMB). Control wells for RPMI 1640 medium and solvent alone were included as negative controls. The positive growth control was composed of fungal inoculum and RPMI-1640. The plates were incubated at 35 °C for 48 h and the reading was performed visually. The MIC value was determined by total growth inhibition as compared to the growth control. Three independent experiments were performed in duplicate.

### 2.4. Human Erythrocyte Hemolysis

The ability of compounds to cause hemolysis was determined by a protocol described and adapted from [14]. Briefly, in a 96-well plate, 50 μL of 2% human erythrocytes suspended in PBS was added to 50 μL of the compounds serially diluted in PBS ranging from 0.25 to 64.0 µg/mL and incubated at 37 °C for 1 hour. Triton X-100 (0.001–1%) was included as a positive control. The plate was then centrifuged at 500× *g* for 5 min and 50 μL of the supernatant from each well of the assay plate was transferred to a fresh 96 well plate. Hemolysis was confirmed by both visual observation and measuring absorbance at 540 nm by a Vmax microplate reader (Molecular Device, Sunnyvale, CA, USA). The assay was conducted in triplicate.

### 2.5. Cytotoxicity Assay

For measuring cytotoxicity, HepG2 cells (ATCC HB 8065; ATCC, Manassas, VA, USA) were cultured in Dulbecco’s modified Eagle medium (DMEM; Life Technologies, Carlsbad, CA, USA) containing 10% fetal bovine serum, 25 mM d-glucose, 2 mM l-glutamine, 1 mM sodium pyruvate and 1% penicillin/streptomycin and maintained at 37 °C in 5% CO_2_. For the toxicity test, HepG2 cells were cultured at 70–80% confluence in 96-well plates in a volume of 100 μL/well culture medium. Serially diluted thiazoles compounds (0.06 to 64 µg/mL) were incubated with the cells at 37 °C in 5% CO_2_ for 24 h. Ten microliters of 2-(4-iodophenyl)-3-(4-nitrophenyl)-5-(2,4-disulfophenyl)-2H-tetrazolium (WST-1) solution (Roche, Mannheim, Germany) was added per well for the last 4 h of the 24 h period. WST-1 reduction was detected using a Vmax microplate reader (Molecular Device, Sunnyvale, CA, USA) to measure absorbance at 490 nm. The percent fluorescence relative to that of the no-treatment control was calculated. The assay was done in triplicate.

### 2.6. Test of Compound Toxicity in G. mellonella

Larvae of the greater wax moth *G. mellonella* were used as an invertebrate model system to evaluate the toxicity of the compounds in a whole organsim. *G. mellonella* larvae (250 to 350 mg; Vanderhorst Wholesale, St. Marys, OH, USA) were injected with 10 μL of each thiazolylhydrazone into the last, left proleg using a micro-syringe (Hamilton, Nevada, USA). For each compound, 10 mg∕kg was injected into a group of 16 larvae. A group injected with PBS was included as a negative control. Larvae were incubated at 37 °C and mortality scored daily. Death was defined as complete loss of mobility and lack of response to physical stimulus using a plastic pipette.

### 2.7. G. mellonella Survival

For this study, the methodology described by [15] was used with some modifications. *G. mellonella* (Vanderhorst Wholesale, St. Marys, OH, USA) in the final larval stage were stored in the dark and used within 7 days from shipment. Prior to *G. mellonella* inoculations, *Candida albicans* CAN14 was grown for 24 h in Yeast Peptone Dextrose (YPD) liquid medium at 30 °C in a shaking incubator. Cells were collected by centrifugation and washed 3 times with PBS and, after this, the number of cells in suspension was determined using a Neubauer chamber (hemocytometer) and the cell number was adjusted to 1.0 × 10^5^.

Groups of 16 larvae randomly chosen with similar weight (250 to 350 mg) and size were used per group in all assays. A hamilton syringe was used to inject 10 µL of the inoculum into the hemocoel of each larva via the last left proleg. After inoculum injection, thiazolylhydrazones compounds (5, 10, or 15 mg/kg) were injected into the last right proleg of the larvae. Fluconazole (16 mg/kg) was administered as a positive control. All antifungal drugs were diluted in PBS. After injection, larvae were incubated at 37 °C, and the number of dead larvae was monitored daily. Two control groups were included; one was inoculated with PBS to observe the killing due to physical trauma, and the other received no injection as a control for general viability.

### 2.8. Murine Models

#### 2.8.1. Animals

C57BL/6 mice, approximately 6–8 weeks in age and 20–25 g were supplied by the Biological Center of the Federal University of Minas Gerais (Cebio, UFMG, Belo Horizonte, Minas Gerais, Brazil). Female mice were used in the oral and systemic candidiasis model. Tests utilizing animal models were in accordance with the Ethics Committee for Animal Experimentation (CEUA/UFMG), protocol number 207/2017.

#### 2.8.2. Oral Candidiasis

The murine model of oral candidiasis was established according to the protocol described previously by [16] with modifications. The antibiotic tetracycline hydrochloride (0.83 mg/mL) was administered in drinking water two days prior to infection, and one day prior to infection the animals were immunosuppressed with subcutaneous injection of prednisolone (100 mg/kg, Amphora, Belo Horizonte, MG, Brazil) and immunosuppression was continued on alternate days until the end of the assay.

For the infection, mice were anesthetized with xylazine hydrochloride (10 mg/kg, Syntec, Hortolândia, São Paulo, Brazil) and ketamine hydrochloride (80 mg/kg, Syntec, Hortolândia, São Paulo, Brazil) via intraperitoneal injection. Anesthetically, the oral cavities were wiped with sterile swabs previously immersed in the inoculum of *C. albicans* CAN14 at a concentration of 10^8^ yeast/mL. A second infection was performed 48 h after the first infection. The next day treatment was started and given every 12 h for 4 days until the animals were sacrificed.

The treatment regimen consisted of 200 μL of compound **2** (100 mg/kg; n = 3), 20 μL of nystatin (600 IU; n = 3, Neoquímica, Anápolis, Goiás, Brazil) or 100 μL of vehicles (as a control) being pipetted into the animal mouths. Compound **1** was diluted in polyethylene glycol 400 (700 μL, 20%, Synth, Diadema, São Paulo, Brazil), Tween 80 (5 μL, 0.05%, Synth, Diadema, São Paulo, Brazil), DMSO (100 μL) and PBS (1200 mL). Mice were sacrificed by cervical dislocation 72 h after the second infection and were immediately observed macroscopically for lesions and the tongues were cleaned with sterile swabs, which were then submerged in 1 mL of sterile PBS. A suspension of 100 μL was plated on CHROMagar *Candida* (Disco, Sparks, MD, USA), and the number of CFUs was enumerated after incubation at 30 °C for 48 h.

#### 2.8.3. Systemic Candidiasis

The murine model of systemic candidiasis was established according to the protocol previously described by [16], with modifications. Mice were infected through the lateral tail vein with 30 μL of *C. albicans* CAN14 cell suspension, resulting in 10^5^ yeasts per animal 3 h prior to the start of antifungal treatment. Treatments to mice, performed intraperitoneally, were started 3 h after infection and persisted every 12 h for 5 days. Animals were treated with fluconazole (10 mg/kg) or compound **2** (10 mg/kg) (n = 6 animals/group). All mice were sacrificed by cervical dislocation after 5 days and the kidneys were removed, observed macroscopically for lesions and colonies, weighed, measured and homogenized in PBS. The homogenates were serially diluted and plated on Sabouraud medium plates. Plates were incubated at 30 °C for 48 h and the fungal load was expressed as the ratio of CFU/g of kidney.

### 2.9. Statistical Analysis

Percent survival and killing curves of *G. mellonella* were plotted and statistical analysis was performed by the Kaplan-Meier test using GraphPad Prism statistical software (GraphPad Software, Inc., California, CA, USA). A *p* value ≤ 0.05 was considered significant. Statistical analysis of results of murine tests used Newman–Keuls Multiple and the Student’s t-test using GraphPad Prism, a *p*-value ≤ 0.05 was considered significant.

## 3. Results and Discussion

### 3.1. Antifungal Susceptibility Tests

Thiazolylhydrazone derivatives included in our study have been previously described [8,9,10]. MICs were determined for four thiazolylhydrazone derivates against reference isolates of several yeasts of clinical interest, in order to evaluate the action spectrum of these substances (Table 1). In general, the tested thiazolylhydrazone had greater efficacy against *C. neoformans*, *C. albicans*, and *C. krusei* than *C. glabrata*. Thiazolylhydrazones compound **1** had the lowest MIC values amongst the most strains in the collection (0.5–32.0 μg/mL), with MICs of 2 μg/mL or below in 7 out of 8 strains, followed by compound **2** whereby 6 out of 8 strains were susceptible at MICs ≤ 2 μg/mL.

As the compounds exhibited activity against the reference isolates, we examined whether these compounds could inhibit clinical isolates of *C. albicans*, non-albicans species, and C. neoformans. Table 2 summarizes the MICs of the four compounds tested for nine clinical isolates of *C. albicans*, for whom the references strains were most susceptible to compounds **1** and **2**. All compounds had antifungal activity against clinical isolates of *C. albicans*. Compounds **1**, **2** and **3** had the lowest MIC values (0.125–2.0 μg/mL); in contrast, compound **4** had the highest MIC values (8.0 to 16.0 μg/mL). Thus, within the group of clinical isolates, compound **3** exhibited slightly more inhibition than was observed against the *C. albicans* reference strains. Fluconazole and amphotericin B had an MIC 90 of 2.0 and 0.25 μg/mL, respectively.

The MIC of the compounds for clinical isolates of *C. parapsilosis* ranged from 1.0 to 4.0 μg/mL and, once again, compound **1** exhibited the most effective activity, with an MIC 90 of 2.0 μg/mL (Table 3). MIC 90 values were 16 μg/mL for fluconazole and 0.5 μg/mL for amphotericin B. According to the cut-off points CLSI M27-S4 [17], MIC values were equal to 4.0 and ≥8.0 μg/mL for *C. parapsilosis* isolates are considered dose-dependent and resistant to fluconazole, respectively. Therefore, in the present study isolates 7449 and 8662 of *C. parapsilosis* showed resistance to fluconazole and, interestingly, the clinical isolates resistant to fluconazole retained susceptibility to the thiazolyhydrazone compounds. Thus, the resistance mechanism of *C. parapsilosis* strains 7449 and 8662 does not convey resistance to the investigational thiazolylhydrazones.

Although the *C. glabrata* reference strains appeared to be resistant to tested thiazolylhydrazone compounds, some clinical isolates exhibited greater susceptibility. Among clinical isolates of *C. glabrata*, we observed that MIC values ranged from 2.0 to 8.0 μg/mL for compound **1**; 2.0 to 16.0 μg/mL for compounds 2 and 3, 2.0 to >32 μg/mL for compound **4** (Table 4). FLC and AMB showed MIC values lower than those found for test compounds and according to CLSI M27-S4 [17], all isolates were sensitive to these drugs.

The antifungal activity of the compounds was also found for clinical isolates of *C. neoformans* (Table 5). Inhibition ranged from 0.25 to 2.0 μg/mL for 1, 2, and 3. Compound **4** (0.5–32.0 μg/mL) exhibited higher MIC values for some *C. neoformans* isolates (MIC 90 = 16 μg/mL).

The evaluation of the MIC of the thiazolylhydrazone derivatives for both clinical and reference isolates showed that these compounds are effective against fungal pathogens, and the best activity can be observed against isolates of *C. albicans* and *C. neoformans*. These results are interesting because *C. albicans* is the main causative agent of candidiasis, the most common fungal infection in humans [3] and *C. neoformans* is a pathogen that causes cryptococcosis in patients with HIV/AIDS, which has limited treatment options due to the relative shortage of potent antifungals available for HIV patients [18]. The results also showed that the thiazolylhydrazone compounds tested have a broad spectrum of action against yeasts of medical interest. Among the tested isolates, compound **1** demonstrated the most consistent low MICs.

### 3.2. Compound Toxicity

**Figure 2:**
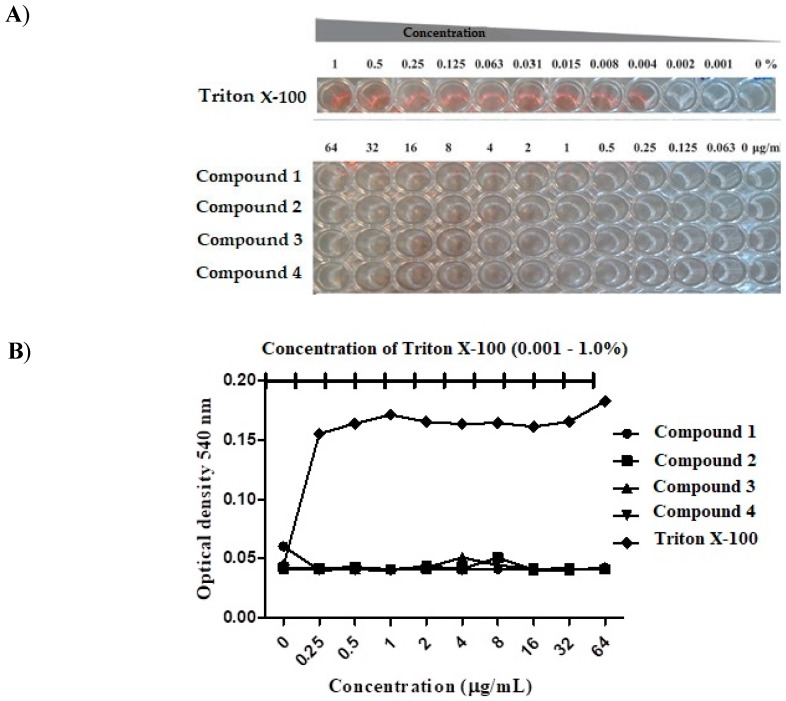
**Hemolytic activity of thiazolylhydrazone.** Human erythrocytes were treated with serial dilutions of the thiazolylhydrazone compounds **1**, **2**, **3** and **4** (0.25–64 μg/mL) or Triton X-100 (0.001–1%). (**A**) Visual inspection of the hemolytic activity demonstrated that the thiazolylhydrazone compounds (**1**–**4**) did not lyse human erythrocytes. The wells in red color indicated that the Triton X-100 lysed the human erythrocytes and acted as a positive control. (**B**) Hemolysis measured spectrophotometrically at 540 nm.

Mammalian erythrocytes represent a good model to evaluate the cytotoxicity of organic and inorganic molecules, natural or synthetic, by the measurement of cellular damage [19]. Therefore, a hemolysis assay was performed using human erythrocytes to assess whether the compounds tested in the present work exhibited any activity against these cells. As observed in Figure 2, none of the compounds tested caused lysis of erythrocytes at the concentrations tested (LC_50_ > 64.0 μg/mL). In contrast, the positive control, Triton X-100, caused hemolysis at all concentrations. These findings are consistent with previous studies showing that thiazoles heterocyclic compounds do not cause damage to mammalian blood cells [20,21].

**Figure 3:**
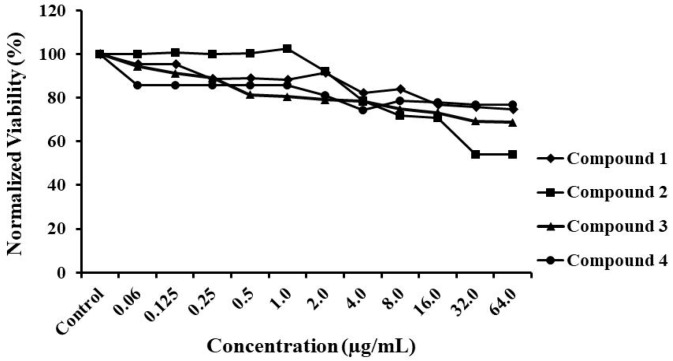
**Thiazolylhydrazones derivatives to HepG2.** The viability of HepG2 cells was measured after treatment with serially diluted concentrations (0.06–64.0 μg/mL) of thiazolylhydrazones derivates. Cell viability was measured spectrophotometrically by detecting degradation of WST-1 dye into formazan by viable cells, which produces an intense color.

A compounds toxicity test was then performed on a human hepatocellular carcinoma (HepG2) cell line that reflects the effect of xenobiotics on the body more than other cell lines, and is one of the most used models for tests of chemical toxicity [22,23]. In this assay, the IC_50_ of the compounds tested ranged from 8.0 to 16.0 μg/mL (Figure 3), eight times higher than the MIC observed for compounds **1** and **2** (0.5 μg/mL) and twice the concentration that 3 and 4 inhibited *C. albicans*. Cells treated with the thiazolylhydrazones derivates were almost 80% viable for compound concentrations up to 8.0 μg/mL and viability dropped with a further increase in concentration. Similar results were previously published describing the low toxicity of compounds **1** and **2** toward human embryonic kidney cells (HEK-293) [10].

### 3.3. G. mellonella Survival

Since the novel antimicrobial compound proved potent in vitro, it was important to evaluate whether this compound was effective against the target microorganism in a model system. In this study, we used *G. mellonella* larvae as an infection model to verify the efficacy of the compounds without adverse toxicity. This invertebrate model allows rapid and effective evaluation of agents in vivo and has significant ethical, logistic, and economic advantages over mammalian models for initial insight into new investigational compounds [24], as well as producing results comparable to those that can be obtained using vertebrate models [25].

**Figure 4:**
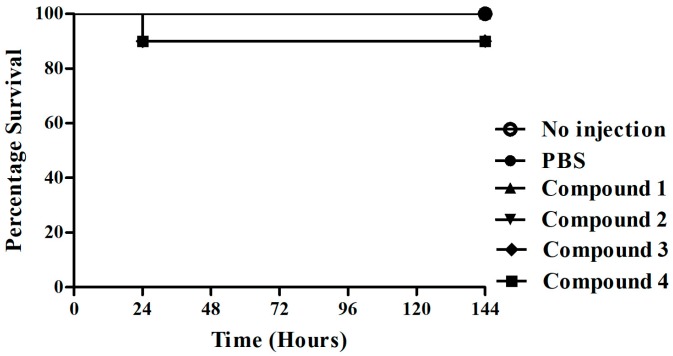
**Toxicity of thiazolylhydrazone in Galleria mellonella.** Larvae were injected with the thiazolylhydrazone compounds **1**, **2**, **3** and **4** at a concentration of 10 mg/kg and their survival was evaluated till 144 h post-treatment. The larvae survival was 100% with the compounds **1** and **2** and 95% with the compounds **3** and **4**.

**Figure 5:**
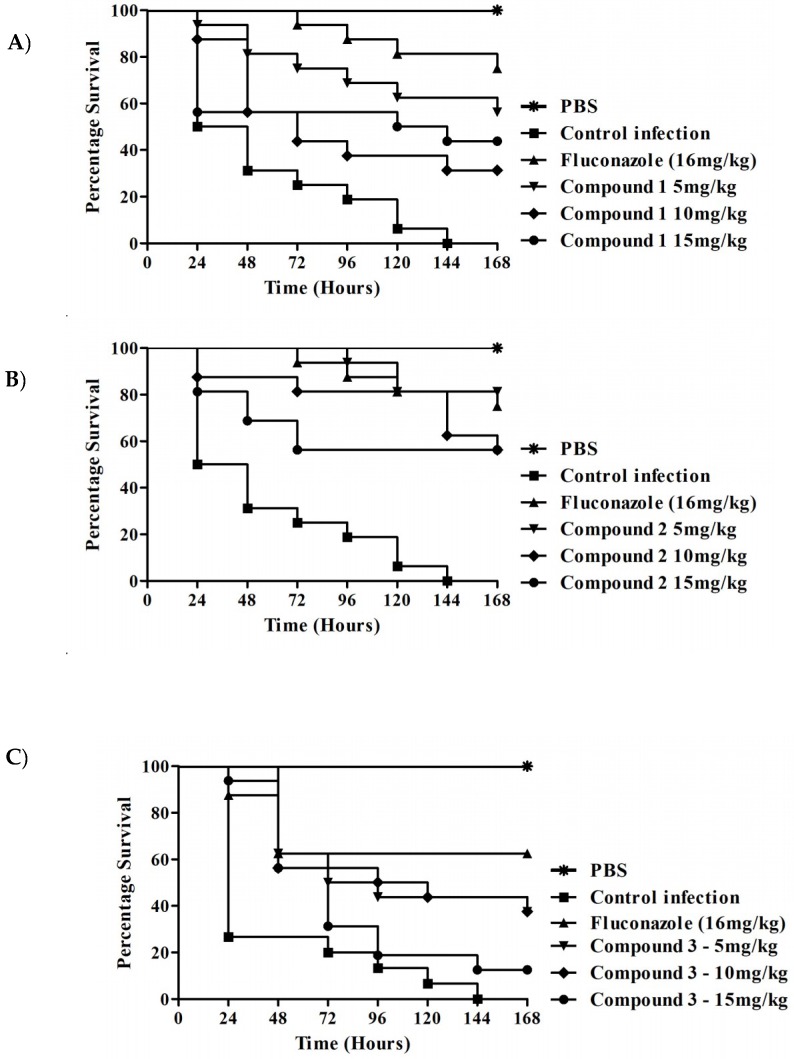

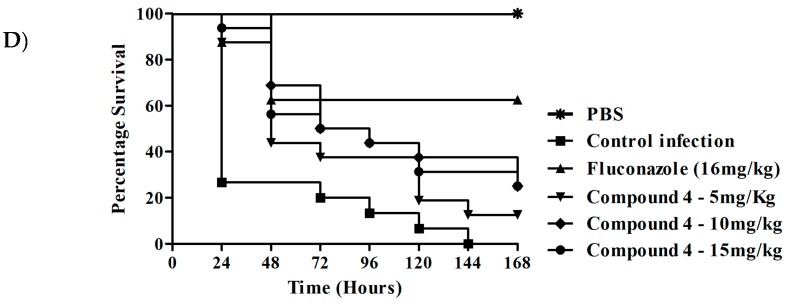
**Survival curve of *Galleria mellonella*.** (**A**) *G. mellonella* infected with *C. albicans* (CAN14) and treated with thiazolylhydrazone **1** at 5.0 mg/kg (*p* < 0.0001); 10 mg/kg (*p* < 0.0128) and 15 mg/Kg (*p* < 0.005) (**B**) *G. mellonella* infected with *C. albicans* (CAN14) and treated with thiazolylhydrazone **2** at 5.0 mg/kg; 10 mg/kg and 15 mg/kg (*p* < 0.0001), (**C**) *G. mellonella* infected with *C. albicans* (CAN14) and treated with thiazolylhydrazone **3** at 5.0 mg/kg (*p* = 0.0005); 10 mg/kg (*p* = 0.0002) and 15 mg/Kg (*p* = 0.0356) (**D**) *G. mellonella* infected with *C. albicans* (CAN14) and treated with thiazolylhydrazone **4** at 5.0 mg/kg (*p* = 0.0224); 10 mg/kg (*p* = 0.0010) and 15 mg/kg (*p* = 0.0026).

Initially, the compound toxicity was carried out in the absence of fungal infection. We observed LD_50_ < 10 mg/kg for all compounds tested. In view of the absence of toxicity, the survival test of the compounds with these larvae was carried out (Figure 4). In the assay of *C. albicans*-infected *G. mellonella* survival, all tested concentrations (5, 10 and 15 mg/kg) of compounds **1**, **2**, **3**, and **4** exhibited a significantly prolonged survival compared to untreated controls (Figure 5). Compounds **1** and **2** demonstrated the highest percentage of live larvae after the seventh day of experiment. For compound **1**, the concentration of 5 mg/kg was the most effective (*p* < 0.0001), showing a survival of 56.3% larvae after the 7th day of treatment. The concentration of 5 mg/kg (*p* < 0.0001) of compound **2** had the best percentage of survival at 81.3%, the other concentrations of 10 mg/kg (*p* < 0.0001) and 15 mg/kg (*p* = 0.0007) presented a 56% increase in survival of the larvae. Compound **3** exhibited survival of only 37% at concentrations of 5 mg/kg (*p* = 0.005) and 10 mg/kg (*p* = 0.005). The highest concentrations of compound **4** at 10 and 15 mg/kg prolonged survival for only 25% of the larvae by the 7th day of the assay.

Overall, the treatment of larvae with the substances under investigation resulted in an increase in larval survival, a similar result was found by [26], which showed a 70% increase in survival of *C. albicans* infected *G. mellonella* larvae at the dose of 5 mg/kg after treatment with CHT, a compound analogous to those used in this work.

### 3.4. In Vivo Assay in Murine Model of Oral and Systemic Candidiasis.

In view of the promising results obtained with compound **2** in the in vitro experiments, where it showed effective activity against *C. albicans* isolates, low cytotoxicity, and increased survival of *G. mellonella* larvae, we further investigated the efficacy of the compound using murine models of oral and systemic candidiasis with this compound at 10 mg/kg.

In the in vivo experiments, we used the oral and systemic candidiasis models because they are the infections most commonly caused by opportunistic pathogenic fungi of the genus Candida present in the indigenous microbiota, which are directly linked to the immunosuppression of the affected individual [27]. In the model of oral candidiasis, a pilot test was performed in the presence and absence of immunosuppression with one and two *C. albicans* inoculations (data not shown). However, animals without immunosupression did not have stable yeasts on the tongue after 5 days and the number of colonies recovered with only one inoculation was very low, so only the experiments with immunosuppressed animals and provision of two infection time points on alternate days are shown.

**Figure 6:**
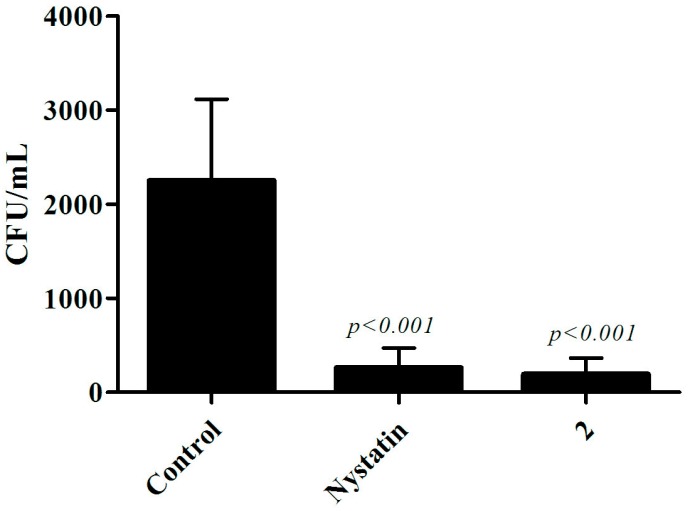
**Murine model treatment of oral candidiasis.** The number of colony-forming units (CFU) recovered from the tongues of C57BL/6 females infected with *C. albicans* CAN14 was lower in the nystatin (600 IU) and thiazolylhydrazone derivative 2 (100 mg/kg) treated animals than in the untreated control group (Newman-Keuls Multiple Comparison test).

After treatment, the animals were sacrificed and the fungal load present on the tongue was evaluated. It can be observed that the animals that received the treatment with compound **2** in this study presented a drastic reduction of the fungal load in relation to the control animals that did not receive any treatment, (*p* < 0.001). The same occurred for nystatin-treated animals (Figure 6). These results show the therapeutic potential of the thiazolylhydrazone derivative 2 in the murine model of oral candidiasis.

Nystatin was used as a control in the oral model because it is the topical agent most commonly used in the treatment of oral candidiasis, presenting better efficacy and lower cost in relation to other existing medications. Because it is not absorbed by the intestine, this antifungal has low potential for side effects, such as vomiting, diarrhea and nausea, adding more advantages [28]. In a study by [16], the authors demonstrated greater or similar efficacy of novel compounds over nystatin. In our study, the compound **2** was able to reduce the fungal load on the tongue of mice similar to nystatin (Figure 6).

**Figure 7:**
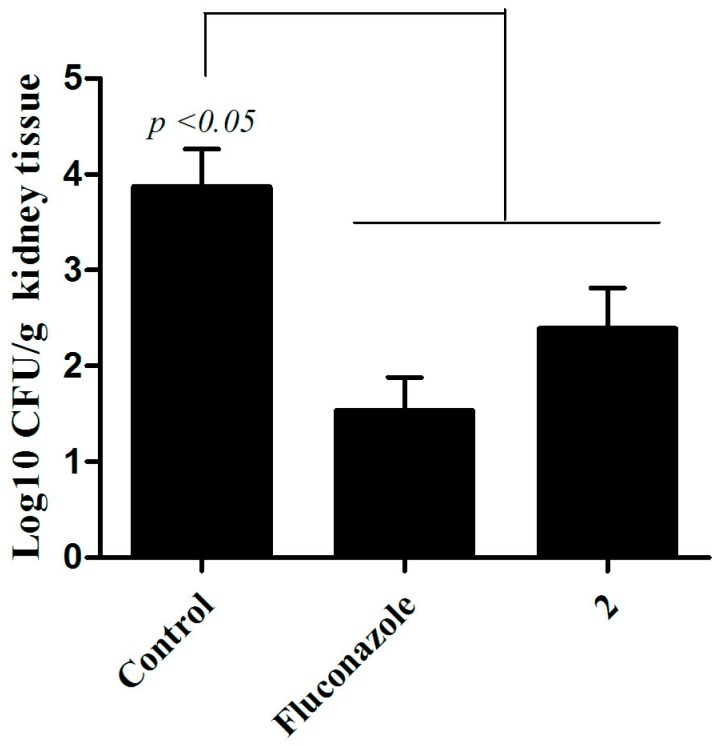
**Murine model treatment of systemic candidiasis.** The number of colony-forming units (CFU) recovered from the *C. albicans* CAN14-infected C57BL/6 female mice was lower in animals treated with fluconazole (10 mg/kg) and thiazolylhydrazone derivative 2 (10 mg/kg) than in the control group that received no treatment (Newman-Keuls Multiple Comparison test).

In the murine model of systemic candidiasis, infection can be found in several organs. However, the kidneys were the organs selected for the analysis, because *C. albicans* typically accumulates in the organs and leads to kidney failure [29,30]. For both fluconazole and compound **2** treatment, a significant reduction (*p* < 0.05) of the fungal load in the kidneys was observed (Figure 7). Thus, by reducing the fungal burden in animals treated with the substance of interest, the results of this assay demonstrate the significant therapeutic potential of the thiazolylhydrazone 2.

## 4. Conclusions

The four thiazolylhydrazone compounds presented antifungal activity against *C. albicans*, and showed activity in vitro against several fungal pathogens, however compounds **1** and **2** presented the best results, with lower toxicity in the cell models studied and were the ones that prolonged the survival of *C. albicans* infected *G. mellonella*. The activity of compound **2** was confirmed in the murine model by reducing the fungal load in the tongue and kidneys of fungal infected animals. Thus, demonstrating the potential of this group of thiazolhydrazone compounds.

## Figures and Tables

**Table 1:** MIC * of thiazolylhydrazones compounds against reference isolates of *Candida* strains and *Cryptococcus neoformans*.

	Thiazolylhydrazones					
Isolate	1	2	3	4	Fluconazole	Amphotericin B
*C. albicans* (DAY-185)	0.5	0.5	4.0	8.0	0.5	1.0
*C. albicans* (CAN14)	1.0	0.5	4.0	4.0	0.125	1.0
*C. krusei* (ATCC6258)	1.0	2.0	2.0	4.0	64.0	8.0
*C. glabrata* (ATCC90030)	32	16.0	32.0	32.0	4.0	2.0
*C. parapsilosis* (ATCC22019)	0.5	2.0	2.0	16.0	8.0	2.0
*C. tropicalis* (ATCC13803)	2.0	8.0	8.0	32.0	>64.0	4.0
*C. dublinensis* (MYA-646)	1.0	2.0	2.0	8.0	1.0	1.0
*C. neoformans* (H99)	0.5	0.5	0.25	2.0	2.0	4.0

* Concentrations in (µg/mL).

**Table 2:** MIC * of thiazolylhydrazones compounds against *Candida albicans* clinical isolates.

	Thiazolylhydrazones					
Isolate	1	2	3	4	Fluconazole	Amphotericin B
02A	1.0	1.0	0.25	8.0	4.0	0.25
02B	0.5	1.0	0.25	8.0	2.0	0.25
7	2.0	2.0	2.0	16.0	1.0	0.125
6	1.0	1.0	0.25	16.0	1.0	0.5
13	0.5	1.0	0.5	8.0	1.0	0.125
1	1.0	1.0	0.5	8.0	1.0	0.25
11	1.0	1.0	0.5	16.0	1.0	0.125
9	1.0	2.0	0.5	16.0	0.5	0.25
10	0.5	1.0	0.5	16.0	0.5	0.25
MIC 50	1.0	1.0	0.5	16.0	1.0	0.25
MIC 90	2.0	2.0	2.0	16.0	2.0	0.25

* Concentrations in µg/mL.

**Table 3:** MIC * of thiazolylhydrazones compounds against *Candida parapsilosis* isolates.

	Thiazolylhydrazones					
Isolate	1	2	3	4	Fluconazole	Amphotericin B
7970A	2.0	4.0	4.0	2.0	1.0	0.25
7652	2.0	2.0	2.0	4.0	4.0	0.25
7449	2.0	4.0	4.0	4.0	16.0	0.5
8662	2.0	4.0	4.0	4.0	16.0	0.25
6901	1.0	4.0	2.0	4.0	0.25	0.5
6917	2.0	4.0	4.0	4.0	0.25	0.25
7839	2.0	4.0	4.0	4.0	0.5	0.5
6933	1.0	4.0	2.0	2.0	0.25	0.5
7585	1.0	4.0	4.0	4.0	0.25	0.25
8044	1.0	2.0	2.0	2.0	0.25	0.5
MIC 50	2.0	4.0	4.0	4.0	0.25	0.25
MIC 90	2.0	4.0	4.0	4.0	16.0	0.5

* Concentrations in µg/mL.

**Table 4:** MIC * of thiazolylhydrazones compounds against *Candida glabrata* clinical isolates.

	Thiazolylhydrazones					
Isolate	1	2	3	4	Fluconazole	Amphotericin B
6922	4.0	16.0	8.0	8.0	2.0	0.5
6927	4.0	8.0	8.0	8.0	2.0	1.0
6931	8.0	16.0	16.0	16.0	0.5	0.5
6932	4.0	8.0	8.0	16.0	0.5	0.5
6943	8.0	16.0	16.0	>32	8.0	1.0
7110	4.0	4.0	4.0	8.0	1.0	0.5
7221	8.0	16.0	16.0	16.0	0.5	0.5
7255	2.0	4.0	4.0	4.0	2.0	1.0
7815	2.0	2.0	2.0	2.0	2.0	0.5
7871	4.0	8.0	8.0	4.0	2.0	1.0
MIC 50	4.0	8.0	8.0	8.0	2.0	0.5
MIC 90	8.0	16.0	16.0	16.0	2.0	1.0

* Concentrations in µg/mL.

**Table 5:** MIC * of thiazolylhydrazones compounds against *Cryptococcus neoformans* clinical isolates.

	Thiazolylhydrazones					
Isolate	1	2	3	4	Fluconazole	Amphotericin B
BF113	1.0	2.0	1.0	16.0	16.0	1.0
BF114	0.5	1.0	1.0	1.0	64.0	<0.0625
41292	0.5	1.0	1.0	1.0	8.0	0.125
41295	1.0	2.0	1.0	8.0	32.0	0.125
41296	2.0	4.0	2.0	32.0	16.0	1.0
41297	1.0	2.0	1.0	16.0	8.0	0.125
41298	1.0	2.0	1.0	8.0	8.0	0.125
41299	0.5	0.5	0.25	4.0	4.0	0.125
C31	0.25	0.25	0.25	8.0	4.0	0.125
F10	1.0	1.0	1.0	4.0	4.0	0.25
RN01	1.0	0.5	0.25	4.0	4.0	0.125
WP	0.5	1.0	0.25	8.0	8.0	1.0
27JF	1.0	2.0	2.0	2.0	8.0	0.25
28JF	0.5	0.5	0.5	8.0	8.0	0.125
90896	1.0	1.0	0.5	8.0	32.0	0.125
93	1.0	1.0	1.0	1.0	4.0	0.25
94	0.25	0.5	1.0	0.5	8.0	0.25
646B	1.0	2.0	2.0	2.0	1.0	0.125
975	1.0	1.0	0.5	16.0	2.0	0.125
9220	0.5	1.0	1.0	1.0	2.0	0.125
9273	0.5	0.5	0.5	0.5	2.0	0.125
10131	0.25	0.5	0.25	0.5	8.0	0.125
10211	0.5	1.0	1.0	1.0	4.0	0.125
10264	0.25	0.5	0.25	0.5	4.0	0.125
10335	0.5	0.5	2.0	0.5	8.0	0.125
10379	0.5	1.0	0.25	8.0	8.0	0.125
92868	0.5	0.5	2.0	0.5	8.0	0.25
MIC 50	0.5	1.0	1.0	4.0	8.0	0.125
MIC 90	1.0	2.0	2.0	16.0	16.0	0.25

* Concentration in µg/mL.
